# Comparative Analysis of Natural Radioactivity Content in Tiles made in Nigeria and Imported Tiles from China

**DOI:** 10.1038/s41598-018-20309-0

**Published:** 2018-01-30

**Authors:** E. S. Joel, O. Maxwell, O. O. Adewoyin, C. O. Ehi-Eromosele, M. A. Saeed

**Affiliations:** 1 Department of Physics, Covenant University Ota, Ota, Nigeria; 2 Department of Chemistry, Covenant University Ota, Ota, Nigeria; 3 Division of Science and Technology, University of Education Township, Lahore, Pakistan

## Abstract

In this investigation, natural radioactive contents in tiles manufactured in Nigeria and tiles imported from China were measured using gamma ray spectroscopy. High Purity Germanium detector was used to estimate the concentrations of some radioisotopes present in 17 samples of various tiles from Nigeria and China. The average activity concentrations of ^226^Ra, ^232^Th, and ^40^K for the tiles were found to be 68.2 ± 0.5; 173.9 ± 9.2 and 490 ± 15 Bq/kg and 58.2 ± 0.5, 161.5 ± 9.4 and 455.7 ± 15.1 Bq/kg for the tiles from Nigeria and China respectively. Radiological hazard indices such as absorbed dose rate, radium equivalent activity, external Hazard Index (H_ex_), internal Hazard Index (H_in_), Annual Effective Dose (mSv/y), Gamma activity Index (Iγ) and Alpha Index (Iα) were determined for both kind of tiles from Nigeria and China. The mean values obtained were: 354.56 and 317.16 Bq/kg; 169.22 nGyh^−1^ and 153.92 nGyh^−1^; 0.95 and 0.87; 1.14 and 1.08; 1.59 mSv/y and 1.52 mSv/y; 1 and 1.15 and; 0.34 and 0.29 respectively. The mean value of radium equivalent obtained in this study is less than that of the international reference value of 370 Bq/kg for the both kind of tiles.

## Introduction

The two major natural sources through which human society are exposed to radiation externally are terrestrial gamma rays and cosmic rays. Terrestrial gamma rays are derivative of natural radionuclides that belongs to ^238^U, ^232^Th and ^40^K series and found in the earth’s crust^1–3^. These radionuclides (^238^U, ^232^Th and ^40^K) are found in soils depending on the distribution of these radionuclides in rocks from which they emanate and on the processes at which the soils are concentrated. Higher radiation levels are generally associated with igneous rocks, such as granite, and lower levels with sedimentary rocks. The specific levels of terrestrial background radiation are related to the types of rocks from which the soils originate^1^. But building materials such as tiles are deliverables from rocks, soil etc which are associated with radioactive material^4,5^. These isotopes occur in all environmental media, but their activity concentrations are generally higher in soils and rocks. But most of these tiles being produced are by-products of rocks and soils which contain various amounts of primordial radionuclides. Enhanced or elevated levels of natural radionuclides in building materials may cause additional doses due to external and internal exposure^6^. The naturally occurring radionuclides in building materials such s tiles contribute to radiation exposure in two ways: the external radiation originating from radiation of the ^238^U and ^232^Th decay series and of ^40^K and the internal radiation due to radon inhalation, leading to deposition of its decay products in the respiratory tract. During past few years, particularly in European, Asian and some African countries attention has been paid to the monitoring of natural radioactivity in building materials^7–23^. These present research work aim to investigate radionuclides concentration and its associated hazard indices in tiles made in Nigeria and compared with tiles imported from China to ascertain the radiation dose level for health safety.

## Materials and Methods

### Preparation of samples

17 samples of various tiles which were produced in Nigeria and China were used for this study. These tiles were purchased from Nigerian commercial market. Tables 1 and 2 give sample names and their ID (size). Initial labeling and cataloguing was done for easy identification. The tiles were broken into smaller pieces so as to allow further processing. All the samples were crushed using the Pascall Engineering Lab milling machine. After each tile sample was crushed, the crusher or lab milling machine was thoroughly cleaned with high pressure blower (Wolf from Kango Wolf power tools, made in London, type 8793 and serial no: 978A) before the next sample was crushed. This whole process was repeated until all the samples were completely crushed into powder. The pulverizer used is the disk ‘grinder/pulverizer’ by Christy & Norris Limited. After each pulverizing process, the machine was cleaned properly and blown with high pressure blower to avoid cross contamination of the samples. The crushed samples were passed through 250 µm sieve mesh size; thereafter1 kg of the sieved sample was weighed out and put in high density polyethylene bottle, well labeled with indelible marker. The samples were transported to Universiti Teknologi Malaysia Nuclear Laboratory for gamma ray analysis. Each sample was weighed again using a digital balance of detection limit of ±0.01 g differently into the Marinelli beakers. The samples were sealed in Marinelli beakers for 4 weeks secular equilibrium for the assumption that the parent nuclide is equal to the daughter. The Marinelli beaker used in this analysis is for both samples and IAEA standard of which they are of the same geometry of the detector size.

**Table 1:** Tiles Samples made in Nigeria.

Sample Name	Country	Size (mm)
BN Ceramics	Nigeria	60 × 60
PNT Ceramics	Nigeria	30 × 30
Golden Crown Ceramics	Nigeria	25 × 30
Royal Ceramics	Nigeria	40 × 40
Royal Crown	Nigeria	30 × 30
Goodwill Super Polish	Nigeria	60 × 60
Goodwill Ceramics	Nigeria	40 × 40
Goodwill Vitrified	Nigeria	40 × 40
PNT Vitrified Tiles	Nigeria	25 × 40
PNT Ceramics	Nigeria	30 × 30

**Table 2:** Tiles Samples Imported from China.

Sample name	Country	Size (mm)
Virony	China	40 × 40
Virony Rustic	China	40 × 40
Virony unglazed	China	30 × 30
Virony Rustic Glass	China	40 × 40
IDDRIS Tiles	China	60 × 60
Virony Glazed	China	40 × 40
Virony Glazed	China	60 × 60

### Gamma ray detection system for the selected samples

The analysis was carried out using the gamma ray spectrometry facilities at the Nuclear Lab. Faculty of Science, Universiti Teknologi Malaysia. A high resolution spectrometer was used for the measurement of the gamma energy spectrum of emitted gamma-rays in the energy range between 50 keV and 2000 keV. The gamma ray spectrometry consists of a high purity germanium (HPGe) detector with a relative efficiency of 20%, a resolution of 1.8 keV for 1332 keV gamma ray emission of ^60^Co. The detector used in gamma ray measurements was Canberra GC2018 with Genie-2000 software. The gamma detector was cooled by liquid nitrogen at 77 K for the purpose of reducing leakage current and thermal noise, and its warm-up sensor is coupled to the high voltage detector bias supply. The pre-amplifier was placed inside a lead shield to reduce background radiation^24^. The Minimum Detectable Activity (MDA) for each radionuclide is U (1 Bq/kg), Th (2 Bq/kg) and K (13 Bq/kg) respectively. The decay isotopes, gamma energy and gamma disintegrations are shown in Table 3.

**Table 3:** The decay Isotopes, Gamma-ray Energy and Gamma disintegration.

Radioactive Series	Decaying Isotopes	Gamma-ray Energy (keV)	Percentage of Gamma Disintegration (%)
Uranium	^214^Pb (for ^238^U decay series)	352.0	35
	^214^Bi (for ^238^U decay series)	609.4	43
Thorium	^208^Tl (for ^232^Th decay series)	583.1	30
	^228^Ac (for ^232^Th decay series)	911.1	29
Potassium	^40^K	1460.8	10.68 or 11

#### Standard sample preparation for gamma spectrometry

The IAEA standard sample Thorium Ore (S-14) and Lake Sediment (SL-2) were used as reference materials and mixed with SiO_2_ in Marinelli beakers. The uranium and Thorium contents from S-14 are 29 ppm and 610 ppm respectively. A weight of 20.00 g from Sample IAEA S-14 was thoroughly mixed with 100.00 g of SiO_2_ in a Marinelli beaker (Coded as S-14). After mixing with SiO_2_, the uranium and thorium concentrations are 4.63 ppm and 97.3 ppm respectively. The IAEA standard sample SL-2 was used to calculate the specific activity of potassium (K). It has a specific activity of 240 Bq kg^−1^. Another Marinelli beaker of 74.18 g of SL-2 was mixed with 100.00 g of SiO_2_ (Coded as SL-2). This provides background for standard samples. The IAEA standard samples used in this study are presented in Table 4.

**Table 4:** IAEA standard samples used in this study.

Standard Sample Code	Concentrations		
	U (ppm)	Th (ppm)	K (Bq kg^−1^)
S-14 (Thorium ore)	29	610	—
SL-2 (Late sediment)	—	—	240

#### Calculation of ^238^U and ^232^Th in Thorium Ore (IAEA S-14) and ^40^K in Lake Sediment (IAEA SL-2)

^**238**^**U:**Concentration of ^238^U in Thorium ore (S-14) = 29 ppm

The weight of measured sample used from S-14 = 20.01 g

The concentration of ^238^U in S-14 used = 29 × 20.01 µg g^−1^ = 580.29 ppm

^**232**^**Th**:

For 232Th in Thorium ore (S-14) = 0.061 (wt. %) = $\frac{0.061\times 100}{1000000}$ = 0.061 × 10^−4^

The measure weight of S-14 used = 20.01 g

The concentration of ^232^Th in S-14 used = 610 × 20.01 = 12206.1 ppm.

^**40**^**K**:

Specific activity of ^40^K = 240 Bq kg^−1^ (IAEA SL-2)

The weight of SL-2 used = 74.18 g

The activity of ^40^K in SL-2 used = $\frac{240Bq}{1000}\times 74.18g$ = 0.24 × 74.18 = 17.8 Bq

#### Measurement of gamma-ray radioactivity from the tiles samples used in this study

The tiles produced in Nigeria and China of different brands were purchased from different suppliers and prepared according to IAEA TRS-295^25^. The samples were sealed and stored for four weeks to achieve secular equilibrium between radium and its progeny^26–29^. Under the conditions of secular equilibrium, ^232^Th concentration was determined from the average concentration of ^208^Tl using the 583 keV peak and ^228^Ac by using the 911 keV peak. ^238^U was determined from the average concentrations of the ^214^Pb by using the 352 keV peak and ^214^Bi by using the 609 keV peak^27–29^. The 1460 keV peak was used to determine the concentration of K. Each sample was put into a shielded HPGe detector and measured for 21600 s. The background gamma-ray spectrum of the detection system was determined with an empty Marinelli beaker under identical conditions, and was subtracted from the spectra of each sample. The activity concentrations were determined by comparing with IAEA standard samples S-14 (Thorium ore) and SL-2 (Lake Sediment). ^238^U activity concentrations were calculated as the arithmetic means of the activities of ^214^Pb, ^214^Bi isotopes and ^208^Tl, ^228^Ac isotopes for ^232^Th. The concentrations of K in (%) was determined from the value obtained in Bq kg^−1^, ^232^Th and ^238^U (ppm) in measured samples were calculated using conversion factors given by^25^.

The concentration of the ^238^U and ^232^Th was determined using Eqs (1) and (2). Equations 3 and 4 was used for ^40^K

1 $${C}_{samp}=\frac{{W}_{std\times {N}_{samp}}}{{W}_{samp\times {N}_{std}}}{C}_{std}$$

where

C_samp_ = concentration of sample collected (ppm).

C_std_ = concentration of the standard sample (ppm). W_std_ = weight of the standard sample (g).

W_samp_ = weight of the sample collected (g).

N_samp_ = net counts of the photopeak area of the sample collected.

N_std_ = net counts of the photopeak area of the standard sample.

The uncertainty of the sample concentration was calculated by using the accurate approach by^28–30^.

2 $${C}_{samp}(ppm)={(\frac{{\rm{\Delta }}{W}_{std}}{{W}_{std}})}^{2}+{(\frac{{\rm{\Delta }}{W}_{samp}}{{W}_{samp}})}^{2}+{(\frac{{\rm{\Delta }}{N}_{samp}}{{W}_{samp}})}^{2}{(\frac{{\rm{\Delta }}{N}_{std}}{{N}_{std}})}^{\frac{1}{2}}\times {C}_{std}$$

Conversion factors were used to convert ppm to Bq kg^−1^. [^238^U; 1 ppm = 12.35 Bq kg^−1^; ^232^Th; 1 ppm = 4.06 Bq kg^−1^]. Whereas 1% of ^40^K = 313 Bq kg^−1^ ^25^.

#### The activity concentration of potassium was calculated by using the formula

 

3 $${A}_{samp=\frac{{W}_{std\times {N}_{samp}}}{{W}_{samp\times {N}_{std}}}{A}_{std}}$$

whereA_samp_ = activity concentration of the sample collected (Bq Kg^−1^).A_std = _activity concentration of standard sample (Bq Kg^−1^).W_std_ = weight of the standard sample (g).W_samp_ = weight of the sample collected (g).N_samp_ = net counts of the photopeak area of the sample collected.N_std_ = net counts of the photopeak area of the standard sample.

The uncertainty of the activity concentration of potassium was calculated by using the following formula^28–30^:

4 $${\rm{\Delta }}{A}_{samp}(ppm)={(\frac{{\rm{\Delta }}{W}_{std}}{{W}_{std}})}^{2}+{(\frac{{\rm{\Delta }}{W}_{samp}}{{W}_{samp}})}^{2}+{(\frac{{\rm{\Delta }}{N}_{samp}}{{W}_{samp}})}^{2}{(\frac{{\rm{\Delta }}{N}_{std}}{{N}_{std}})}^{\frac{1}{2}}\times {A}_{std}$$

## Results and Discussions

### Determination of activity concentration

The radioactivity concentration evaluated in the tiles made in Nigeria and China is presented in Tables 5 and 6. The observed activities concentration of the radionuclides content in the tiles from Nigeria and China ranged from 27 ± 0.5–241 ± 0.5 and 42.5 ± 0.5–75.0 ± 0.5 Bq/kg for ^226^Ra, 41 ± 9.4–461 ± 9.5 and 41.5 ± 9.4–405.5 ± 9.4 Bq/kg for ^232^Th, and 270 ± 15–860 ± 15 and 290 ± 15.1–740 ± 15 Bq/kg for ^40^K respectively. The results show that PNT ceramic tiles (30 × 30 mm) made in Nigeria and Virony Glazed tiles (40 × 40 mm) imported from China had the highest ^226^Ra concentration of 241 ± 0.5 and 75 ± 0.5 Bq/kg, respectively; NISPRO and Virony Rustic Glass tiles of size 40 × 40 mm have 461 ± 9.5 and 405 ± 9.4 Bq/kg for ^232^Th and 860 ± 15 Bq/kg for ^40^K while Iddris tile imported from China has value of 740 ± 15 Bq/kg for ^40^K respectively. The lowest values of 27 ± 0.5 and 42.5 ± 0.5, 41 ± 9.4 and 41.5 ± 9.4, and 270 ± 15 and 290 ± 15.1 Bq/kg are found for tiles samples BN ceramics and Virony Rustic Glass tile, Royal crown and Virony Rustic, and Goodwill super polish and Virony Glazed. The mean value concentration of the radionuclides ^226^Ra, ^232^Th, and ^40^K for both tiles made in Nigeria and imported tiles from china are 68.2 ± 0.5 and 58.2 ± 0.5, 173.9 ± 9.2 and 161.5 ± 9.4, and 490 ± 15 and 455.7 ± 15.1 Bq/kg respectively. These mean values were found to be within international reference value when compared with^31^ report but from the present study, it was observed that tiles produced in Nigeria have higher radionuclides concentration.

**Table 5:** Radioactivity concentration in tiles made in Nigeria in (Bq/kg).

Sample Name	Sample size	Activity Concentration (Bq/kg)		
		^226^Ra	^232^Th	^40^K
BN Ceramics	60 × 60	37.5 ± 0.5	101.5 ± 9.1	670.0 ± 15.0
PNT Ceramics	30 × 30	241.0 ± 0.5	77.5 ± 9.1	510.0 ± 15.0
Golden Ceramics	25 × 40	49.5 ± 0.5	57.5 ± 8.4	460.0 ± 15.0
Royal Ceramics	40 × 40	65.5 ± 0.6	44.0 ± 9.3	390.0 ± 15.1
Royal Crown	30 × 30	51.5 ± 0.5	41.0 ± 9.4	440.0 ± 15.1
Goodwill Super Polish	60 × 60	44.0 ± 0.5	51.5 ± 9.3	270.0 ± 15.0
NISPRO	40 × 40	59.5 ± 0.6	461.0 ± 9.5	860.0 ± 15.0
Goodwill Vitrified	40 × 40	70.5 ± 0.5	445.5 ± 9.5	540.0 ± 15.1
PNT Vitrified	25 × 40	35.5 ± 0.5	346.5 ± 9.4	370.0 ± 15.0
Golden Crown	30 × 30	27.0 ± 0.5	113.0 ± 8.9	390.0 ± 15.0
Mean values		68.2 ± 0.5	173.9 ± 9.2	490.0 ± 15.0

**Table 6:** Radioactivity concentration in tiles imported from China in (Bq/kg).

Sample name	Size	Activity Concentration (Bq/kg)		
		^226^Ra	^232^Th	^40^K
Virony	40 × 40	55.5 ± 0.5	126.5 ± 9.5	530.0 ± 15.1
Virony Rustic	40 × 40	59.5 ± 0.5	41.5 ± 9.4	480.0 ± 15.0
Virony unglazed	30 × 30	55.0 ± 0.5	52.0 ± 9.4	440.0 ± 15.1
Virony Rustic Glass	40 × 40	42.5 ± 0.5	63.0 ± 9.3	390.0 ± 15.1
IDDRIS Tiles	60 × 60	65.0 ± 0.5	337.0 ± 9.4	740.0 ± 15.0
Virony Glazed	40 × 40	75.0 ± 0.5	405.5 ± 9.4	290.0 ± 15.1
Virony Glazed	60 × 60	55.0 ± 0.5	105.0 ± 9.7	320.0 ± 15.1
Mean values		58.2 ± 0.5	161.5 ± 9.4	455.7 ± 15.1

### Hazard indices assessment

#### The absorbed dose rate

In this study, the absorbed dose rates obtained from the calculated activity concentrations are shown in Tables 7 and 8. The total air absorbed dose rate received in an open air 1 m above the ground due to gamma emission from the radionuclides of ^226^Ra, ^232^Th and ^40^K in BqKg^−1^ available in an environment is calculated using Eq. (5)^1,12^

5 $${\rm{D}}({{\rm{nGyh}}}^{-1})=0{{\rm{.642C}}}_{{\rm{Ra}}}+0{{\rm{.604C}}}_{{\rm{Th}}}+0{{\rm{.0417C}}}_{{\rm{k}}}{ < \text{80nGyh}}^{-1}$$

**Table 7:** Radium Equivalent (Raeq), The Absorbed Dose, External Hazard Index (Hex) and Internal Hazard Index (Hin) for the tiles made in Nigeria.

Sample Name	Sample size	Ra_eq_(Bq/kg)	D(nGyh^−1^)	H_ex_	H_in_
BN Ceramics	60 × 60	234. 24	113.32	0.63	0.73
PNT Ceramics	30 × 30	391.09	222.79	1.06	1.71
Golden Crown Ceramics	25 × 30	167.15	85.69	0.45	0.59
Royal Ceramics	40 × 40	158.45	84.89	0.43	0.61
Royal Crown	30 × 30	144.01	76.18	0.39	0.53
Goodwill Super Polish	60 × 60	138.44	70.61	0.37	0.49
NISPRO	40 × 40	784.95	352.51	2.11	2.28
Goodwill Vitrified	40 × 40	749.15	336.86	2.02	2.21
PNT Ceramics	25 × 40	559.49	247.51	1.51	1.61
Golden Crown	30 × 30	218.62	101.85	0.53	0.66
Mean values		354.56	169.22	0.95	1.14

**Table 8:** Radium Equivalent (Raeq), The Absorbed Dose, External Hazard Index (Hex) and Internal Hazard Index (Hin) for the tiles imported from China.

Sample Name	Sample size	Ra_eq_(Bq/kg)	D(nGyh^−1^)	H_ex_	H_in_
Virony	40 × 40	227. 21	134.14	0.74	0.89
Virony Rustic	40 × 40	155.81	83.28	0.42	0.92
Virony unglazed	30 × 30	163.24	85.07	0.44	0.59
Virony Rustic Glass	40 × 40	162.62	81.60	0.44	0.55
IDDRIS Tiles	60 × 60	603.89	276.14	1.63	1.81
Virony Glazed	40 × 40	677. 9	305.17	1.82	2.03
Virony Glazed	60 × 60	229.79	112.07	0.62	0.77
Mean values		317. 16	153.92	0.87	1.08

Considering the absorbed dose rates presented in Tables 7 and 8, it can be observed that the highest value of 352.51 and 305.17nGyh^−1^ was reported in NISPRO and Virony Glazed tiles whereas the lowest value of 70.61 and 81.60 nGyh^−1^ was noted in Goodwill super polish and Virony Rustic Glass tile respectively. Comparing the average absorbed dose rate in this present study with the standard value of 80 nGyh^−1^ recommended by^12^, the highest value obtained in this present study is higher by a factor of 4.4 and 3.7 respectively for both locally produced tile in Nigeria and imported tiles from China.

#### Radium equivalent (Raeq) determination

The level of radionuclides from ^226^Ra, ^232^Th and ^40^K in the analyzed building materials is non- uniformly distributed. The Raeq activity of the measured radionuclides is used to compare the activity of each of ^226^Ra, ^232^Th and ^40^K contents in the building materials such as tiles. Raeq with unit as BqKg^−1^ was calculated using Eq. (6).

6 $${\text{Raeq}=\text{AC}}_{{\rm{RA}}}+1{{\rm{.43AC}}}_{{\rm{Th}}}+0{{\rm{.077AC}}}_{{\rm{K}}}$$

where AC_RA_, AC_Th_ and AC_K_ are the activities concentration of ^226^Ra, ^232^Th and ^40^K measured in BqKg^−1^ respectively. This radium equivalent activity defines the weighted sum of the individual activities of ^226^Ra, ^232^Th and ^40^K with the idea that for ^226^Ra, Raeq is 10 Bq kg^−1^, for ^232^Th, Raeq is 7 Bq kg^−1^ and for ^40^K, Raeq is 130 Bqkg^−1^. The maximum value of Raeq in tiles materials must be less than 370 Bq kg^−1^ as recommended by^1^ and^12^. The radium equivalent activity values obtained from this present study varies from 138.44 to 784.95 BqKg^−1^ with the highest value of 784.95 BqKg^−1^ reported in NISPRO whereas the lowest value of 138.44 BqKg^−1^ is noted in Goodwill super polish tile and the mean value of 354.56 BqKg^−1^ is noted. It can be observed that some tiles samples such as PNT Ceramics (30 × 30), NISPRO, Goodwill Vitrified and PNT Ceramics (25 × 40) have the Raeq value that exceeds the recommended limit of 370 BqKg^−1^ by^1^ and^12^ as presented in Table 7. The radium equivalent for the tiles imported from China varies from 155.81 to 677.9 BqKg^−1^ with the Virony Glazed having the highest value of 667.9 BqKg^−1^ while Virony Rustic reported to have the lowest value of 155.81 BqKg^−1^. The average value of radium equivalent is 317.16 BqKg^−1^ and is within the recommended international reference value of 370 BqKg^−1^. This is shown in Table 8.

#### Evaluation of external hazard index

The gamma ray radiation hazards index due to the specified radionuclides were assessed by external radiation hazard and was calculated using Eq. (7) according to^1^.

7 $${{\rm{H}}}_{{\rm{ex}}}=({{\rm{C}}}_{{\rm{Ra}}}/370)+({{\rm{C}}}_{{\rm{Th}}}/259)+({{\rm{C}}}_{{\rm{K}}}/4810)$$

where, C_Ra_, C_Th_ and C_K_ are the average activity concentrations of ^226^Ra, ^232^Th and ^40^K in Bq kg^−1^ respectively. For the radiation hazard to be acceptable, it is recommended that the H_ex_ be less than unity. The estimated H_ex_ for all the tile samples produced varies from 0.37 to 2.11 with highest value noted in NISPRO tile whereas the lowest value reported in Goodwill super polish as shown in Table 7. This highest value from the present study is higher than the recommended value of ≤1 according to^1^. The estimated external hazard index for the imported tiles from China varies from 0.42 to 1.82 with highest value observed in Virony Glazed tile of size 40 × 40 mm whereas the lowest value was reported in Virony Rustic tile. The estimated highest value for the H_ex_ of the tiles from Nigeria and China are 2.11 and 1.82 respectively. This is presented in Table 8.

#### Determination of internal hazard index

The hazard which is defined in relation to internal hazard is represented by H_in_ respectively and can be found using Eq. (8)^32^:

8 $${{\rm{H}}}_{{\rm{in}}}=({{\rm{C}}}_{{\rm{Ra}}}/185)+({{\rm{C}}}_{{\rm{Th}}}/259)+({{\rm{C}}}_{{\rm{K}}}/4810)$$

where C_Ra_, C_Th_ and C_K_ are activity concentrations of ^226^Ra, ^232^Th and ^40^K, respectively in Bq/kg. For the safe use of a building material such as tiles for decorative purposes in construction, H_in_ should be less than unity. The calculated values of H_in_ for tile samples used are shown in Tables 7 and 8. The values ranged between 0.53 and 2.28 and the mean values of 1.14 for internal hazard (H_in_) for the tiles samples produced in Nigeria. The obtained results for H_in_ for PNT ceramic, NISPRO, Goodwill super polish and PNT ceramic (25 × 40) tiles which are produced in Nigeria are above recommended limit of unity. The results for other tile samples are less than unity and are in agreement with the recommended international values. For the tiles imported from China, the estimated values for the sample are shown in Table 8. Its value ranged between 0.55 and 2.03 with a mean value of 1.08. The result obtained for internal hazard for the IDDRIS Tiles and Virony Glazed are higher than recommended limit of 1 for the China tiles as well as the tiles produced in Nigeria.

#### The annual effective dose rate

The indoors annual effective dose equivalent received by human is estimated from the indoor internal dose rate (Din), occupancy factor which is defined as the level of human occupancy in an area in proximity with radiation source; is given as 80% of 8760 h in a year, and the conversion factor of 0.7 Sv Gy^−1^ which is used to convert the absorbed does in air to effective dose^1^. The annual effective dose equivalent is estimated using Eq. (9).

9 $${\rm{AED}}{\rm{R}}=(0\text{.49CRa}+0\text{.76CRTh}+0\text{.048CK})\times 8{.76\times 10}^{-3}$$

The value of the AEDE for the tiles produced in Nigeria and tiles imported from China ranges from 0.65 to 3.69 mSv y^−1^ with a mean value of 1.59 mSv y^−1^ and 0.73 to 3.14 mSv y^−1^ with an average value of 1.52 respectively. The mean values from the samples (tiles made in Nigeria and imported tiles from China) surpass the world’s average value of 0.07 mSv y^−1^. Details of annual effective dose rate for all the samples are presented in Tables 9 and 10.

**Table 9:** The Annual Effective Dose (mSv/y), γ -activity Index (Iγ) and Alpha Index for the tiles made in Nigeria.

Sample Name	Sample size	Annual Effective Dose (mSv/y)	Gamma activity Index(Iγ)	Alpha Index (Iα)
BN Ceramics	60 × 60	1.12	0.76	0. 19
PNT Ceramics	30 × 30	1.76	1.36	1.21
Golden Crown Ceramics	25 × 30	0.79	0.61	0.25
Royal Ceramics	40 × 40	0.74	0.57	0.33
Royal Crown	30 × 30	0.68	0.52	0.26
Goodwill Super Polish	60 × 60	0.65	0.49	0.22
NISPRO	40 × 40	3.69	2.79	0.29
Goodwill Vitrified	40 × 40	2.84	0. 18	0.35
PNT Ceramics	25 × 40	2.61	1.97	0. 18
Golden Crown	30 × 30	1.03	0.79	0. 14
Mean values		1.59	1.00	0.34

**Table 10:** The Annual Effective Dose (mSv/y), γ -activity Index (Iγ) and Alpha Index for the tiles imported from China.

Sample Name	Sample size	Annual Effective Dose (mSv/y)	Gamma activity Index(Iγ)	Alpha Index (Iα)
Virony	40 × 40	1.30	0.99	0.28
Virony Rustic	40 × 40	0.73	0.57	0.29
Virony unglazed	30 × 30	0.77	0.59	0.28
Virony Rustic Glass	40 × 40	0.77	0.59	0.21
IDDRIS Tiles	60 × 60	2.83	2.14	0.33
Virony Glazed	40 × 40	3.14	2.37	0.38
Virony Glazed	60 × 60	1.07	0.82	0.28
Mean value		1.52	1.15	0.29

#### Gamma index determination (Iγ)

Gamma index is used to evaluate the **γ**-radiation hazard related to the natural radionuclide in the particular samples under investigation. The gamma index representation (Iγ) is estimated using Eq. (10) as presented by^33^.

10 $${\rm{I}}\gamma ={{\rm{C}}}_{{\rm{Ra}}}/300({{\rm{Bqkg}}}^{-1})+{{\rm{C}}}_{{\rm{Th}}}/200({{\rm{Bqkg}}}^{-1})+{{\rm{C}}}_{{\rm{K}}}/3000({{\rm{Bqkg}}}^{-1})$$

The estimated results are presented in Tables 9 and 10. The controls on the radioactivity of building materials according to RP122^34^ is based on the dose criterion for control and exemption. The dose effective that is above the criterion level of 1 mSvy^−1^ should be taken into consideration for radiation protection. It recommends that controls of dose range of 0.3 to 1 mSvy^−1^, which is the excess gamma dose to that received outdoors. The gamma activity index is used to identify whether a dose criterion is met^34^. This gamma activity index accounts for the ways and amounts in which the materials used in building, with limit value of their indices not exceeding the recommended value and depends on the dose criterion shown in Tables 9 and 10. In this present investigation, the dose has been evaluated excluding the background dose which was shielded by the building materials when used in bulk but does not still exclude when building materials used as a superficial material. This is because the thin layers of superficial material do not reduce significantly the background dose. The gamma activity index ≤1, corresponds to annual effective dose less than or equal to 1 mSvy^−1^, while gamma activity index ≤0.5 corresponds to 0.3 mSvy^−1^ if the materials are used in bulk quantity. At the same time, gamma activity index ≤6 corresponds to annual effective dose of 1 mSvy^−1^ and gamma activity index ≤2 corresponds to an annual effective dose ≤0.3 mSvy^−1^ if the bulk materials are used in a superficial way. In this study as shown in Tables 9 and 10 respectively, the results for superficial materials such as tiles which is made in Nigeria vary from 0.18 (Goodwill vitrified tile) to 2.79 (NISPRO tile) with average value of 1.00 while for the imported tiles from China, the gamma index value range from 0.57 (Virony Rustic) to 2.37 (Virony Glazed of size 40 × 40 mm). When using building materials such as tile, it should be exempted from all restrictions regarding radioactivity if the excess gamma radiation emanating from them increases the annual effective dose of a member public by 0.3 mSv at the most. Considering the criterion of unity that corresponds to annual effective of 1 mSv, all the present values are below the criterion which corresponds to the protection level except PNT ceramic (30 × 30), NISPRO and PNT ceramic (25 × 40) tiles which were produced in Nigeria as well IDDRIS tile and Virony Glazed of size 40 × 40 mm being imported from China.

#### Determination of alpha index (Iα)

The assessment of the alpha index is another important aspect of hazard assessment that deals with the estimation of that excess alpha radiation due to radon inhalation originating from building materials. The alpha index calculated using Eq. (11)^35,36^ is:

11 $${{\rm{I}}{\rm{\alpha }}={\rm{C}}}_{{\rm{Ra}}}{/200(\text{Bqkg}}^{-1})$$

where C_Ra_ is the activity concentration of radium Bqkg^−1^ in building materials. If the radium activity level in building material exceeds the values of 200 Bqkg^−1^ there is possibility that the radon exhalation from the material could cause indoor radon concentrations exceeding Bqm^−3^. Tables 9 and 10 presents the values for alpha index. The International Commission on Radiation protection recommends an action level of 200 Bqm^−3^ for radon in dwellings^37^. At the same time, if this radium activity level is below 100 Bqkg^−1^, it shows that radon exhalation from building materials may not likely cause indoor concentration greater than 200 Bqm^−3^ ^36^. It is reported that the recommended exempted value and the recommended upper limit for radon concentrations are 100 Bqkg^−1^ and 200 Bqkg^−1^ respectively in building materials^38^. It is noted that the upper limit of radon concentration (Iα) is equal to 1^39^. The results of the present study show that the radon concentration varies from 0.14 to 1.21 respectively with average value of 0.34 for the tile produced in Nigeria while for imported tiles from China; it varies from 0.21 to 0.33 with mean value of 0.29.

## Conclusions

The measurement of natural radioactivity concentrations and its associated radiological risks have been evaluated in 17 types of different tile samples from Nigeria and China for building purposes. The mean activity concentration of ^226^Ra, ^232^Th and ^40^K have been found to be 68.2 ± 0.5; 173.9 ± 9.2 and 490 ± 15 Bq/kg for tiles produced in Nigeria, while that of imported from China are 58.2 ± 0.5, 161.5 ± 9.4 and 455.7 ± 15.1 Bq/kg respectively. On the average, activity concentration of ^226^Ra, ^232^Th and ^40^K were lower than the international recommended value. The radium equivalent activity for most of the tiles samples used is less than the recommended value of 370 Bq/kg set in by UNSCEAR^1^ report excluding PNT ceramic (30 × 30), NISPRO, Goodwill Vitrified, PNT Ceramics (25 × 40) tiles with a value of 391. 10, 784.95, 749.15 and 559.49 Bq/kg and IDDRIS tile, Virony Glazed and Virony Glazed (40 × 40 mm) have values of 603.89 and 677.9 Bq/kg respectively. The absorbed dose rate in air was found to be in the ranged of 70.61 to 352.51 nGyh^−1^ with mean value of 169.22 nGyh^−1^ which is higher than international value of 55 nGyh^−1^ by factors of 3.2 and 2.1 according to UNSCEAR^12^ and 80 nGyh^−1^ by^1^ while tiles imported from China ranged between 81.60 nGyh^−1^ and 305.17 nGyh^−1^ and higher than the recommended value as established by^1^ and^12^. The average value of H_ex_ and H_in_ are 0.95 and 1.14 respectively. The mean value of H_ex_ is lower than unity as recommended by^1^ while H_in_ is higher. It was also observed that PNT ceramic (30 × 30), NISPRO, Goodwill Vitrified and PNT Ceramics (25 × 40) have values higher that international reference value. However, for the imported tiles from China, the mean value of H_ex_ and H_in_ are 0.87 and 1.08 except IDDRIS tiles, all other tile samples imported from China are within the recommended limit of unity. The result of annual effective dose rate show a higher value in tile samples BN ceramic, PNT ceramic (30 × 30 mm), BN ceramic, NISPRO, Goodwill Vitrified, and PNT ceramic (25 × 40) for tiles produced in Nigeria and IDDRIS tile, Virony Glazed (40 × 40 mm) and Virony Glazed (60 × 60 mm) are above recommended value of 1 mSv/yr as well as on the average value. The mean values of gamma activity index for the tiles made in Nigeria and imported tiles from China are 1.00 and 1.15 respectively and is still within the world recommended value except for the tiles imported from China and the Alpha Index (Iα) are 0.34 and 0.29. The present results indicate that the tiles sample such as PNT ceramic (30 × 30), NISPRO, Goodwill Vitrified, PNT Ceramics (25 × 40), IDDRIS tile, Virony Glazed (40 × 40 mm) and Virony Glazed (60 × 60 mm) should be monitored before using in construction of building. The higher values of gamma index activity observed in some samples such as PNT ceramic (30 × 30), NISPRO, Goodwill Vitrified, PNT Ceramics (25 × 40), IDDRIS tile, Virony Glazed (40 × 40 mm) and Virony Glazed (60 × 60 mm) from Nigeria and China could be attributed to the nature of the geological sources of these samples which serves as raw material for the production of the tiles.

## Electronic supplementary material

Supplementary information

## References

[1] UNSCEAR. Sources, Effects, & Risks of Ionizing Radiation. Report to the General Assembly, with Scientific Annexes, *UN*, *New York*, **I & II**, 2–47 (2000).

[2] Murty VRK, Karunakara N (2008). Natural radioactivity in the soil samples of Botswana. Radiat. Meas..

[3] Psichoudaki M, Papaefthymiou H (2008). Natural radioactivity measurements in the city of Ptolemais (Northern Greece). J. Environ. Radioact..

[4] Baykara O, Karatepe S, Dogru M (2011). Assessments of natural radioactivity and radiological hazards in construction materials used in Elazig, Turkey. Radiat. Meas..

[5] Lu X, Yang G, Ren C (2012). Natural radioactivity and radiological hazards of building materials in Xianyang, China. Radiat. Phys. Chem..

[6] Marocchi M, Righi S, Bargossi GM, Gasparotto G (2011). Natural Radionuclides Content and Radiological Hazard of Commercial Ornamental Stones: An Integrated Radiometric and Mineralogical -Petrographic Study. Radiat. Meas..

[7] Arafa W (2004). Specific activity and hazard of granite samples collected from the Eastern desert of Egypt. J. Environ. Radioact..

[8] Malanca A, Pessina V, Dallara G, Luce NC, Gaidolfi L (1995). Natural radioactivity in building materials from Brazilian state of Espirito Santo. Appl. Radiat. Isot..

[9] Giuseppe C, Garavaglia M, Magnoni S, Viali G, Vecchi R (1996). Natural radioactivity and radon exhalation rate in stony materials. J. Environ. Radioact..

[10] Petropoulos NP, Anagnostakis MJ, Simopoulos SE (2002). Photon attenuation, natural radioactivity content and radon exhalation rate of building materials. J. Environ. Radioact..

[11] Ahmad MN, Hussein AJA (1997). Natural radioactivity in Jordanian building materials and the associated radiation hazards. J. Environ. Radioact..

[12] UNSCEAR. Sources, Effects and Risks of Ionizing Radiations. *United Nations*, *New York*, 1–7 (1998).

[13] Muhammad I, Muhammad T, Sikander MM (2001). Measurement of natural radioactivity in marbles found in Pakistan using a NaI(Tl) gamma ray spectrometer. J. Environ. Radioact..

[14] Sroor A, El Bahi SM, Ahmed F, Abdel Haleem AS (2001). Natural radioactivity and radon exhalation rate of soil in southern Egypt. Appl. Radiat. Isot..

[15] Kovler K, Haquin G, Manasherov V, Ne’eman E, Lavi N (2002). Natural radionuclides in building materials available in Israel. Build. Environ..

[16] Stoulos S, Manolopoulo M, Papastefanou C (2003). Assessment of natural radiation exposure and radon exhalation from building materials in Greece. J. Environ. Radioact..

[17] Rahaman S, Matiullah, Mujahid AS, Hussain S (2008). Assessment of radiological hazards due to the presence of natural radionuclides in samples of building materials collected from the northwestern areas of Pakistan. J. Radiol. Prot..

[18] Sharaf JM, Hamideen MS (2013). Measurement of natural radioactivity in Jordanian building materials and their contribution to the public indoor amma dose rate. Appl. Radiat. Isot..

[19] Al-Jarallah MI, Abu-Jarad F, Fazal-ur-Rehman MI (2001). Determination of radon exhalation rate from tiles using active and passive techniques. Radiat. Meas..

[20] Tufan MÇ, Dişci T (2013). Natural radioactivity measurements in building materials used in Samsun, Turkey. Radiat. Prot. Dosim.

[21] Amin SA, Naji M (2014). Natural radioactivity in different commercial ceramic samples used in Yemeni buildings. Radia. Phy. and Chem..

[22] Sharma N, Singh J, Esakki SC, Tripathi RM (2016). A study of the natural radioactivity and radon exhalation rate in some cement used in India and its radiological significance. J. Radia. Res. and Appl. Sci...

[23] El-Dine W, El-Shershaby A, Ahmed F, Abdel-Haleem AS (2001). Measurement of radioactivity and radon exhalation rate in different kinds of marbles and granites. Appl. Radiat. Isot..

[24] Tsoulfanidis, N. Measurement and Detection of Radiation. Taylor and Francis, 2 edition, New York (1995).

[25] IAEA. Technical Reports, Series No: 295, International Atomic Energy Agency. (1989)

[26] Alnour I (2012). Natural radioactivity measurements in the granite rock of quarry sites, Johor, Malaysia. Radia. Phy. and Chem..

[27] Ibrahim NM, Abd El Ghani AH, Shawky SM, Ashraf EM, Faruk MA (1993). Measurement of radioactivity level in soil in Nile Delta and Middle Egypt. H. Phy..

[28] Maxwell O, Wagiran H, Ibrahim N, Lee SK, Soheil S (2013). Comparison of ^238^U ^232^Th,and ^40^K in different layers of subsurface structuresion Dei-Dei and Kubwa, Abuja, Northcentral Nigeria. Radia. Phy. and Chem..

[29] Maxwell O, Wagiran H, Ibrahim N, Lee SK, Soheil S (2013). Measurement of ^238^U, ^232^Th, and ^40^K in boreholes at Gosa and Lugbe, Abuja, North Central Nigeria. Radia. Pro. Dosim..

[30] Supian, B. S. & Evans, C. J. Statistics and nuclear counting: theory, problems and solutions Statistics and errors in measurements, 26–35 (1992).

[31] International Atomic Energy Agency. Extent of Environmental Contamination by Naturally Occurring Radioactive Material (NORM) and Technological Options for Mitigation, Technical Reports Series No. 419, STI/DOC/010/419 (2003).

[32] Beretka J, Mathew PJ (1985). Natural radioactivity of Australian building materials, industrial waste and byproducts. H. Phys..

[33] OECD (Organization for Economic Co- operation and Development). Exposure to radiation from radioactivity in building materials. Report by a group of experts of the OECD Nuclear Energy Agency (1979).

[34] EC (1999). Radiological protection principles concerning the natural radioactivity of building materials. Radia. Prot..

[35] Righi S, Bruzzi L (2006). Natural radioactivity and radon exhalation in building materials used in Italian dwellings. J. Environ. Radioact..

[36] ICRP (1994). Protection against Rn-222 at home and at work. ICRP publication 65. Ann ICRP..

[37] Xinwei L, Lingqing W, Xiaodan J, Leipeng Y, Gelian D (2006). Specific activity and hazards of Archeozoic–Cambrian rock samples collected from the Weibei area of Shaanxi, China. Radiat. Prot. Dosim..

[38] RPA. Naturally occurring radiation in the Nordic countries; Recommendations. Stockholm: Statens stralskyddsinstitut, 15–63 (2000).

[39] Tufail M, Nasim A, Sabiha J, Tehsin H (2007). Natural radioactivity hazards of building bricks fabricated from soil of two districts of Pakistan. J. Radio. Prot..

