# Supplementary material for: Comparative Analysis of Natural Radioactivity Content in Tiles made in Nigeria and Imported Tiles from China
Source: Sci Rep. 2018 Jan 30;8:1842. doi: 10.1038/s41598-018-20309-0 (PMC5789825; doi:10.1038/s41598-018-20309-0)
Supplement: Supplementary file 1 — Supplementary information [file 41598_2018_20309_MOESM1_ESM.pdf]

Comparative Analysis of Natural Radioactivity Content in Tiles Made in Nigeria and Imported Tiles from China

E.S Joel[1*], O. Maxwell[1], O.O Adewoyin[1], C.O Ehi-eromesele[2], M. A Saeed[3]

		Procedure for calculating error values								
MB	KeV	d Wstd	W std	d Wsam	Wsam	d Nsam	Nsam	d Nstd	Nstd	Cstd
PNT Ceramics (30X 30mm)	352	59.72	580.145	0.01	532.6	159.97	4140	60.78	1630	4.72
	609	59.73	580.145	0.01	532.6	132.58	4900	46.16	1340	4.72
	583	854.21	12203.05	0.01	532.6	132.1	2250	74.04	4320	99.2
	911	854.21	12203.05	0.01	532.6	115.94	2180	56.96	2670	99.2
	1460	2.15	17.8	0.01	5.33	102.86	7340	40.42	1420	120
MB	KeV	d Wstd	W std	d Wsam	Wsam	d Nsam	Nsam	d Nstd	Nstd	Cstd
Golden Ceramics (25X 40mm)	352	59.72	580.145	0.01	467.3	139.83	4140	60.78	1630	4.72
	609	59.73	580.145	0.01	467.3	112.56	4900	46.16	1340	4.72
	583	854.21	12203.05	0.01	467.3	112.43	2250	74.04	4320	99.2
	911	854.21	12203.05	0.01	467.3	81.92	2180	56.96	2670	99.2
	1460	2.15	17.8	0.01	4.67	102.82	7340	40.42	1420	120
MB	KeV	d Wstd	W std	d Wsam	Wsam	d Nsam	Nsam	d Nstd	Nstd	Cstd
Royal Ceramics (40X 40mm)	352	59.72	580.145	0.01	573.9	180.29	4140	60.78	1630	4.72
	609	59.73	580.145	0.01	573.9	159.28	4900	46.16	1340	4.72
	583	854.21	12203.05	0.01	573.9	145.23	2250	74.04	4320	99.2
	911	854.21	12203.05	0.01	573.9	116.34	2180	56.96	2670	99.2
	1460	2.15	17.8	0.01	5.74	152.87	7340	40.42	1420	120

d Cstd	Cstd Avg.	d Cstd avg	
0.55	4.72	0.54	
0.53			
9.22	99.20	9.10	
8.97			
14.99	120	14.99	
d Cstd	Cstd Avg.	d Cstd avg	
0.54	4.72	0.53	
0.52			
8.70	99.20	8.43	
8.16			
14.99	120	14.99	
d Cstd	Cstd Avg.	d Cstd avg	
0.56	4.72	0.55	
0.53			
9.60	99.20	9.29	
8.98			
15.10	120	15.10	
